# Quantification of collagen and proteoglycan deposition in a murine model of airway remodelling

**DOI:** 10.1186/1465-9921-6-30

**Published:** 2005-04-08

**Authors:** Alistair K Reinhardt, Stephen E Bottoms, Geoffrey J Laurent, Robin J McAnulty

**Affiliations:** 1 Centre for Respiratory Research, University College London, Rayne Building, 5 University Street, London WC1E 6JJ, UK

## Abstract

**Background:**

Sub-epithelial extracellular matrix deposition is a feature of asthmatic airway remodelling associated with severity of disease, decline in lung function and airway hyperresponsiveness. The composition of, and mechanisms leading to, this increase in subepithelial matrix, and its importance in the pathogenesis of asthma are unclear. This is partly due to limitations of the current models and techniques to assess airway remodelling.

**Methods:**

In this study we used a modified murine model of ovalbumin sensitisation and challenge to reproduce features of airway remodelling, including a sustained increase in sub-epithelial matrix deposition. In addition, we have established techniques to accurately and specifically measure changes in sub-epithelial matrix deposition, using histochemical and immunohistochemical staining in conjunction with digital image analysis, and applied these to the measurement of collagen and proteoglycans.

**Results:**

24 hours after final ovalbumin challenge, changes similar to those associated with acute asthma were observed, including inflammatory cell infiltration, epithelial cell shedding and goblet cell hyperplasia. Effects were restricted to the bronchial and peribronchial regions with parenchymal lung of ovalbumin sensitised and challenged mice appearing histologically normal. By 12 days, the acute inflammatory changes had largely resolved and increased sub-epithelial staining for collagen and proteoglycans was observed. Quantitative digital image analysis confirmed the increased deposition of sub-epithelial collagen (33%, p < 0.01) and proteoglycans (32%, p < 0.05), including decorin (66%, p < 0.01). In addition, the increase in sub-epithelial collagen deposition was maintained for at least 28 days (48%, p < 0.001).

**Conclusion:**

This animal model reproduces many of the features of airway remodelling found in asthma and allows accurate and reproducible measurement of sub-epithelial extra-cellular matrix deposition. As far as we are aware, this is the first demonstration of increased sub-epithelial proteoglycan deposition in an animal model of airway remodelling. This model will be useful for measurement of other matrix components, as well as for assessment of the molecular mechanisms contributing to, and agents to modulate airway remodelling.

## Background

Asthma is a chronic disease with increasing prevalence characterised by persistent bronchial hyperreactivity [1]. There is evidence of epithelial cell damage, inflammatory cell infiltration, together with hypertrophy and hyperplasia of goblet cells, submucosal glands and airway smooth muscle. Additionally, there is increased deposition of extracellular matrix molecules including collagen types I [2], III [3-5] and V [3,5-7], fibronectin [2], tenascin [5,8] and the proteoglycans, lumican, biglycan, versican and decorin [9,10] in the sub-epithelial *lamina reticularis*. This leads to a 2-3-fold increase in the thickness of the *lamina reticularis *[11,12] that has been correlated with increased fibroblast/myofibroblast number [11].

The importance of increased airway extracellular matrix deposition in the pathogenesis of asthma is uncertain. However, increasing evidence suggests it may play a significant role. Severity of asthma and decline in FEV_1 _correlate with sub-epithelial fibrosis [5,13,14]. Airway hyperresponsiveness correlates with bronchial wall thickening [15], sub-epithelial fibrosis [5,7,13], airway wall proteoglycan immunoreactivity [9] and airway fibroblast proteoglycan production [16]. In addition, mathematical modelling suggests that thickening of the sub-epithelial layer will result in an altered folding pattern of the airway with fewer circumferential folds and an increased tendency to airway obstruction [17]. The mechanisms responsible for increased sub-epithelial extracellular matrix deposition in asthma are unknown, although they are thought to involve a complex interaction between cells and mediators in the airway wall [18,19]. Furthermore, the effects of current treatments on this aspect of the pathology are equivocal [6,20-24].

Further studies are therefore required to determine the precise mechanisms involved in the sub-epithelial deposition of extracellular matrix proteins, its importance in relation to airway function and the effects of existing and new therapeutic agents on the process. Mechanistic studies of airway remodelling are difficult in man. Consequently, there is a need for good, reproducible animal models of airway remodelling and techniques to assess these parameters, preferably in mice, which would allow study of genetically-modified strains. Increased sub-epithelial deposition of collagen has been demonstrated in mouse models [25-28], but there is little or no information on other extra-cellular matrix proteins. The sensitivity of mice to airway remodelling is variable [29,30] and protocols to achieve sustained airway remodelling can take several months [28]. In addition, the methods used to assess sub-epithelial matrix deposition are either qualitative [31] or, where quantitative methods have been used, they are often subjective, laborious [25,32], or not specific to measurement of airway changes [33].

In this study we have used C57Bl6/SV129 mice, a strain commonly used in the generation of genetically-modified animals, to develop a model which exhibits many characteristics of asthmatic airways and produces a rapid, reproducible and persistent increase in sub-epithelial extracellular matrix. In conjunction with this we developed accurate and sensitive histological methods using computer-assisted image analysis to specifically quantitate changes in sub-epithelial collagen and proteoglycan deposition.

## Methods

### Ovalbumin sensitisation and challenge

All animal studies were undertaken with appropriate local ethical and government regulatory approval. SV129/C57BL/6 mice were bred at University College London from breeding pairs obtained from the Jackson Laboratory, Bar Harbor, ME. Two to three month old mice were sensitised by intra-peritoneal injection of 10 μg chicken ovalbumin (grade V, Sigma, Poole, U.K.) in 0.1 ml normal saline or saline alone on two occasions 10 days apart. Twenty-one days after the second sensitisation, mice were challenged with six daily intratracheal instillations of 400 μg ovalbumin in 50 μl normal saline or saline alone. Briefly, mice were anaesthetised using an intra-peritoneal injection of 0.1 ml Saffan™ anaesthetic (9 mg alfaxalone and 3 mg alfadolone acetate/ml, Schering-Plough Animal Health, Welwyn Garden City, U.K.). An intra-venous cannula (22G/25 mm, BOC Ohmeda AB, Helsingborg, Sweden) was introduced through the mouth into the trachea and ovalbumin was instilled using a Hamilton syringe passed through the lumen of the cannula. Sham challenged mice received normal saline. Mice were killed in groups either one or twelve days after the final challenge to assess acute airway changes and airway remodelling respectively. Animals were injected intra-peritoneally with 0.1 ml Euthatal (200 mg pentobarbitone sodium/ml, Rhône Mérieux Ltd., Harlow, U.K.). A longitudinal ventral incision was made in the abdomen and the major vessels were sectioned. Lungs were removed and either wax embedded or frozen as described below.

### Lung tissue preparation for histochemical and immunohistochemical staining of paraffin wax sections

Insufflation of the bronchial tree was performed using tracheal instillation of 4% paraformaldehyde at 20 cm 4% paraformaldehyde. The trachea was then ligated and the thoracic contents were removed. Lungs were fixed in 4% paraformaldehyde at 4°C overnight and then transferred to 15% sucrose in PBS at 4°C overnight. They were rinsed in 50% ethanol/H_2_O and stored in 70% ethanol/H_2_O prior to further dehydration and processing using an automated vacuum tissue processor (Leica TP1050, Leica Microsystems (U.K.) Ltd., Milton Keynes, U.K.). Lungs were embedded in paraffin wax using an embedding unit (Blockmaster III, Raymond A. Lamb, London, U.K.). Transverse 3 μm sections were cut for histochemical staining and 5 μm sections for immunohistochemistry. After sacrifice, care was taken to perform instillation at a constant pressure of paraformaldehyde. Lungs from each experiment were processed together in precisely the same way and sections were cut and stained at the same time. Automated staining was also pivotal to the uniform production of stained sections for image analysis.

### Lung tissue preparation for proteoglycan staining of frozen sections

Insufflation of the bronchial tree was performed using tracheal instillation of a 1 : 3 mixture of OCT embedding matrix (Cell Path plc, Newtown, U.K.) : PBS at a pressure of 25 cm of this mixture. Due to the increased viscosity of this mixture, compared with paraformaldehyde, a higher pressure was required to attain the same degree of inflation. The trachea was ligated and thoracic contents were removed. Lungs were transferred to 50 ml polypropylene tubes containing OCT embedding matrix and the tubes were placed into a container of iso-pentane, chilled using liquid nitrogen. Frozen sections were cut at a thickness of 7 μm, placed on polylysine-coated slides and stored at -20°C before staining.

### Histochemical staining

Paraffin wax embedded sections (3 μm) were stained with haematoxylin and eosin to demonstrate tissue architecture and inflammatory cell infiltration, and para-amino salicylic acid/Alcian blue to demonstrate mucous cells. A modified Martius Scarlet Blue (MSB) trichrome stain was employed for the localisation and quantification of collagen using computerised image analysis.

### Immunohistochemistry

Immunohistochemistry for type III collagen and decorin was performed on paraffin embedded sections (5 μm). Sections were dewaxed, rehydrated and antigen retrieval achieved by either proteinase K digestion (10 μg/ml) for 10 minutes at room temperature for type III collagen or by microwaving sections in 10 mM citrate buffer, pH6, for 10 min for decorin. Sections were washed in PBS and endogenous peroxidase activity was blocked with 3% hydrogen peroxide (Sigma, Poole, U.K.) for 30 min at RT. Sections were then washed in PBS and incubated with 4% (v/v) goat serum (DakoCytomation, Ely, U.K.) for 20 min at RT to block non-specific binding sites. Excess serum was removed by blotting and the sections were incubated with either a 1 : 200 dilution of rabbit anti-human type III collagen antibody (Chemicon, Chandlers Ford, UK) or a 1 : 80 000 dilution of Decorin antibody, LF-113 [34] (kindly provided by Dr L.W. Fisher, National Institutes of Health, Bethesda, MA. U.S.A.), overnight at 4°C. The sections were washed and incubated with 0.5% (v/v) goat anti-rabbit biotin-labelled secondary antibody (DakoCytomation, Ely, U.K.) for 60 min at RT. Further washes in PBS were performed before the sections were incubated with 0.5% (v/v) streptavidin (DakoCytomation, Ely, U.K.) for 30 min at RT. The streptavidin was removed by three washes in TBS and the sections were treated with DAB for 10 min. They were then washed in tap water, counterstained in Mayer's haematoxylin, differentiated in acid alcohol, dehydrated and mounted.

### Proteoglycan Staining

Frozen sections (7 μm) were stained with Cupromeronic Blue™ (Seikagaku Corporation, Tokyo, Japan) using critical electrolyte concentration (CEC) methodology [38]. Cupromeronic Blue is an intensely coloured and electron-dense cationic dye used for the detection of sulphated polyanions including proteoglycans [36]. Sections were rinsed for 5 min in distilled water and placed into the staining solution overnight. This consisted of 0.05% Cupromeronic Blue in 25 mM sodium acetate buffer (pH 5.8) containing 2.5% glutaraldehyde and 250 mM magnesium chloride. Different CEC were trialled before 250 mM of magnesium chloride was selected because it provided optimal staining. This CEC is appropriate for detection of small leucine-rich proteoglycans such as decorin (J.E. Scott, personal communication). The next day slides were washed in 0.025 M sodium acetate buffer (pH 5.8) containing 250 mM magnesium chloride. They were dehydrated to xylene and coverslips were applied.

### Measurement of airway sub-epithelial matrix components using image analysis

Sections were examined by light microscopy using a x10 objective. Airways were selected using the following pre-defined criteria. Suitable airways were: complete, of an appropriate size to be contained within a high power field, not attached to other airways and cut in a plane perpendicular to their length (the minimum internal diameter : maximum internal diameter ratio was more than 0.5 in all cases). All suitable airways in each section were analysed. Images were digitised using a digital video camera (JVC KY-F55B, Imaging Associates, Thame, U.K.) with a resolution of 768 × 576 (vertical × horizontal) pixels. Pixel size was converted into micrometers and image analysis was performed using image analysis software (Zeiss KS300 Release 3.0, Imaging Associates, Thame, U.K.). Thresholding using pre-defined RGB criteria for airspace was performed. This allowed lumen airspace to be differentiated from airway wall. Airway lumen perimeter was then measured for each suitable airway. Thresholding using predefined RGB criteria for extra-cellular matrix constituents was performed. This produces a superimposed digitised image of both airway and parenchymal matrix. Next, a 'scrapping' procedure was completed in which pixels not adjoining at least ten other pixels were deleted. This provided better definition of airway matrix as links between airway and parenchymal matrix were removed. In this way, lumen perimeter and matrix values were obtained for each airway. Results were expressed as area of sub-epithelial matrix/unit airway perimeter (μm^2^/μm).

### Statistical analysis

Data are presented as means ± SEM. Statistical evaluations were performed using ANOVA or unpaired t-tests for single group comparisons. A p-value of less than 0.05 was considered significant.

## Results

Many of the histological changes associated with airway remodelling in asthma are reproduced in this murine model following ovalbumin sensitisation and intra-tracheal challenge. Characteristic differences were seen one and twelve days after final challenge.

### Histological changes seen 24 hours after the final challenge

**Figure 1:**
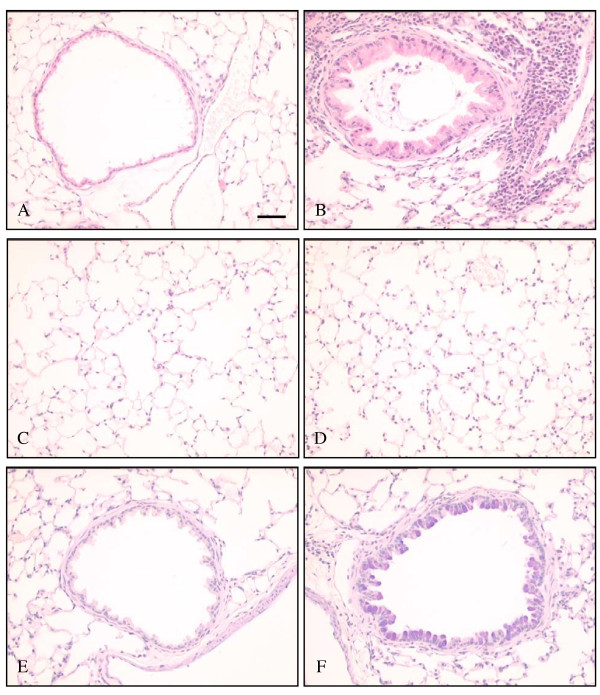
**Histological appearance of murine lung 24 hours following final ovalbumin challenge. **H&E staining of **(A) **saline sensitised/challenged and **(B) **ovalbumin sensitised/challenged airways at 24 hours after final challenge. Control airways did not show any inflammatory cell infiltration. A mixed inflammatory cell infiltrate can be seen around ovalbumin sensitised/challenged airways, particularly in those adjacent to blood vessels where margination and diapedesis of cells are seen. H&E staining of typical areas of parenchyma from **(C) **saline sensitised/challenged and **(D) **ovalbumin sensitised/challenged mice are similar, demonstrating the lack of interstitial inflammation produced by this model. PAS/Alcian Blue staining of **(E) **saline sensitised/challenged and **(F) **ovalbumin sensitised/challenged airways at 24 hours after final challenge. Most of the epithelial cells are replaced by blue/purple staining goblet cells in ovalbumin sensitised/challenged airways. Scale bar represents 50 μm.

Airway changes on the day after the final challenge are shown in figure 1. H&E-stained sections from saline sensitised/saline challenged mice demonstrated that the epithelial layer was generally intact and epithelial cells appeared regular in shape (figure 1A). Inflammatory cell infiltration of the airway wall was absent. Very occasional mucus-secreting goblet cells were demonstrated using PAS/Alcian Blue staining (figure 1E). Lung sections from saline sensitised/ovalbumin challenged and ovalbumin sensitised/saline challenged mice were not distinguishable from those of saline sensitised/challenged mice (data not shown). H&E staining of sections from ovalbumin sensitised/challenged mice revealed widespread disruption of the epithelial layer (figure 1B). Epithelial cells were irregular in shape, some were pyknotic and some were shed into the airway lumen. A large number of mucus-secreting goblet cells were demonstrated using PAS/Alcian Blue staining (figure 1F). Airway walls were infiltrated with inflammatory cells that were predominantly eosinophilic but also included neutrophils, lymphocytes and macrophages (figure 1B). Margination and diapedesis of inflammatory cells was seen in adjacent blood vessels (figure 1B). Inflammatory cell infiltration was restricted to the bronchial and peribronchial regions. The lung parenchyma appeared to be unaffected by ovalbumin sensitisation and challenge, and was histologically similar to control sections (figure 1C, D).

### Histological changes seen 12 days after the final challenge

#### Epithelial changes

**Figure 2:**
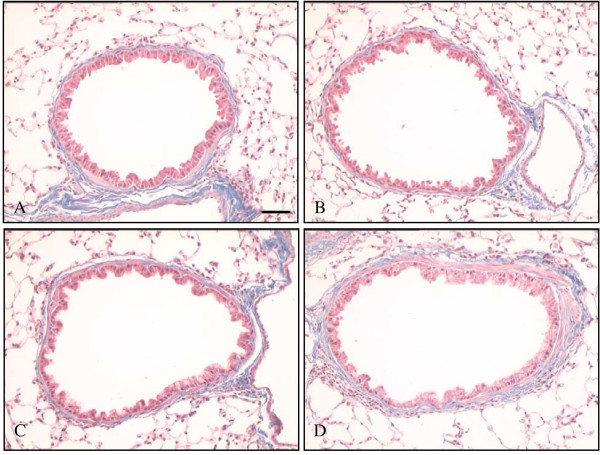
**Increased sub-epithelial collagen deposition in the airways of ovalbumin sensitised and challenged mice. **MSB staining of representative airways from each treatment group at 12 days after final challenge. **(A) **saline sensitisation/saline challenge **(B) **saline sensitisation/ovalbumin challenge **(C) **ovalbumin sensitisation/saline challenge **(D) **ovalbumin sensitisation/ovalbumin challenge. Increased blue-staining sub-epithelial collagen is seen in the ovalbumin sensitised/challenged airway compared with the three controls, which have similar appearances. Scale bar represents 50 μm.

Inflammatory changes were no longer apparent in ovalbumin sensitised/challenged airways and were not seen in any of the control groups (figure 2). The epithelial layer was generally intact and epithelial cells appeared regular in shape. Very occasional mucus-secreting goblet cells were demonstrated using PAS/Alcian Blue staining (data not shown). All three control groups had similar appearances (figure 2A and 2C).

#### Increased sub-epithelial collagen deposition

**Figure 3:**
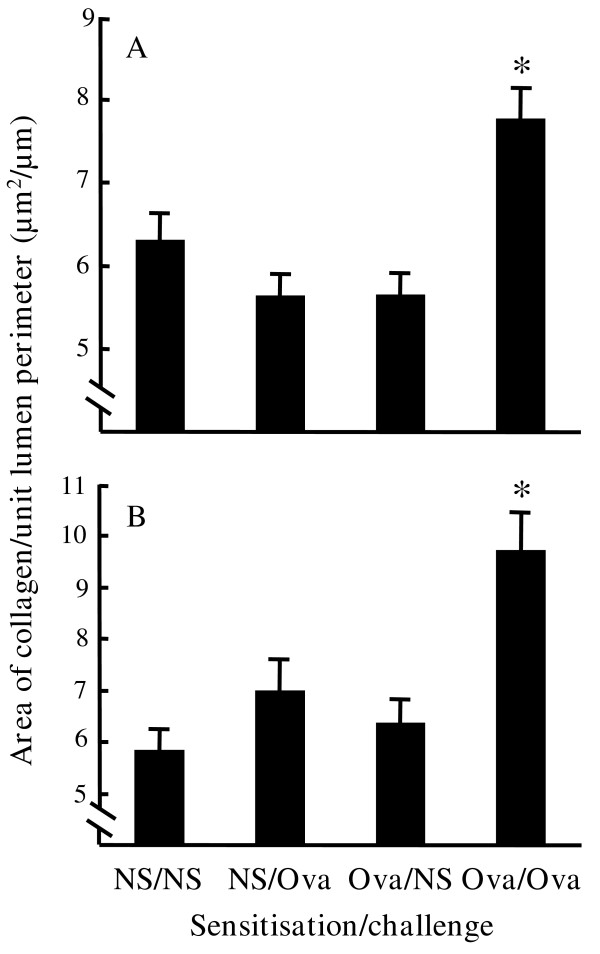
**The effect of different sensitisation/challenge combinations on sub-epithelial collagen deposition. **The area of sub-epithelial collagen/unit lumen perimeter was quantitated in mouse airways using image analysis of MSB stained sections. **(A) **Mean values for all suitable airways in each experimental group were compared. The numbers of mice/total airways analysed per group were: saline sensitised/saline challenged (NS/NS) 8/89, saline sensitised/ovalbumin challenged (NS/Ova) 9/95, ovalbumin sensitised/saline challenged (Ova/NS) 7/88 and ovalbumin sensitised/ovalbumin challenged (Ova/Ova) 11/147. **(B) **Mean values for all suitable larger (perimeter>1000 μm) airways in the same mice were also compared. The numbers of airways analysed were NS/NS 48, NS/Ova 30, Ova/NS 22 and Ova/Ova 39. * p < 0.01 compared with the mean of the controls.

In control sections, MSB staining showed a diffuse band of blue-stained collagen surrounding the airways beneath the epithelial layer (figure 2A2B2C). In smaller airways the band of collagen sometimes appeared discontinuous. However, in the airways of ovalbumin sensitised and challenged mice, the staining appeared more intense, the band of staining was thickened compared with controls and usually appeared continuous around all sizes of airway (compare figure 2D with figures 2A2B2C). Quantitative image analysis showed there were no significant differences in the area of sub-epithelial collagen staining between the control groups (figure 3A). Values for control groups were 6.31 ± 0.32 μm^2^/μm for saline sensitised/challenged mice, 5.63 ± 0.27 μm^2^/μm for saline sensitised/ovalbumin challenged mice and 5.65 ± 0.26 μm^2^/μm for ovalbumin sensitised/saline challenged mice. Ovalbumin sensitisation and challenge produced a significant increase in sub-epithelial collagen/unit lumen perimeter compared with control groups (7.77 ± 0.37 μm^2^/μm, p < 0.01). Compared with the mean of the controls, there was a 33% increase in sub-epithelial collagen in ovalbumin sensitised/challenged animals (p < 0.01).

In these studies the area of sub-epithelial collagen staining was expressed with respect to length of airway lumen perimeter. Therefore differences could reflect changes in lumen perimeter as well as collagen deposition. However, we found no significant difference in lumen perimeter between control groups (991 ± 40 μm) and the ovalbumin sensitised/ovalbumin challenged group (1066 ± 55 μm) indicating that the changes observed were directly attributable to an increase in sub-epithelial collagen deposition.

To determine whether ovalbumin sensitisation and challenge had a differential effect on sub-epithelial collagen depending on airway size, airway data were sub-divided into lumen perimeters of greater or less than 1000 μm (figure 3B). In airways with lumen perimeters greater than 1000 μm, values for control groups were 5.84 ± 0.42 μm^2^/μm for saline sensitised/challenged mice, 7.00 ± 0.61 μm^2^/μm for saline sensitised/ovalbumin challenged mice and 6.36 ± 0.48 μm^2^/μm for ovalbumin sensitised/saline challenged mice. There were no significant differences between these groups. Sub-epithelial collagen/unit lumen perimeter was 9.73 ± 0.73 μm^2^/μm in ovalbumin sensitised/challenged mice, representing a 53% increase in collagen compared with the mean of the controls (p < 0.01). Ovalbumin sensitisation and challenge did not appear to increase the area of sub-epithelial collagen compared with saline sensitisation and challenge in airways with a lumen perimeter less than 1000 μm. Values for control groups were 6.63 ± 0.55 μm^2^/μm for saline sensitised/challenged mice (number of airways analysed, n = 43), 5.03 ± 0.25 μm^2^/μm for saline sensitised/ovalbumin challenged mice (n = 66) and 5.19 ± 0.31 μm^2^/μm for ovalbumin sensitised/saline challenged mice (n = 50). Sub-epithelial collagen/unit lumen perimeter was 6.58 ± 0.40 μm^2^/μm in ovalbumin sensitised/challenged mice (n = 88).

In a further experiment sub-epithelial collagen was measured 28 days after final challenge in order to assess whether the increase in collagen persisted over time. Saline sensitised/saline challenged mice were compared with ovalbumin sensitised/ovalbumin challenged mice. Only one control group was used as no significant differences had been shown between control groups in the previous experiment. Sub-epithelial collagen/unit lumen perimeter was 3.69 ± 0.22 μm^2^/μm in saline sensitised/saline challenged mice and 5.46 ± 0.42 μm^2^/μm in ovalbumin sensitised/challenged mice, a 48% increase in collagen (p < 0.001). In airways with lumen perimeters greater than 1000 μm, values for saline sensitised/challenged mice were 4.43 ± 0.35 μm^2^/μm (n = 34) and 6.92 ± 0.79 μm^2^/μm (n = 21) in ovalbumin sensitised/challenged mice, corresponding to a 56% increase in collagen (p < 0.002) and similar to that observed at 12 days. In contrast to the 12-day data, in airways with lumen perimeters less than 1000 μm, values for saline sensitised/challenged mice were 2.88 ± 0.16 μm^2^/μm (n = 31) and 4.58 ± 0.43 μm^2^/μm (n = 35) in ovalbuminsensitised/challenged mice, corresponding to a 59% increase in collagen (p < 0.001). The increase in sub-epithelial collagen is therefore maintained for at least four weeks.

Sections from two groups at 12 days were compared using the same techniques by an independent observer. Sub-epithelial collagen/unit lumen perimeter was 4.16 ± 0.21 μm^2^/μm in saline sensitised/saline challenged mice and 5.65 ± 0.24 μm^2^/μm in ovalbumin sensitised/challenged mice. This represents an increase of 36% (p < 0.001) and is of a similar magnitude to that obtained by the first observer.

**Figure 4:**
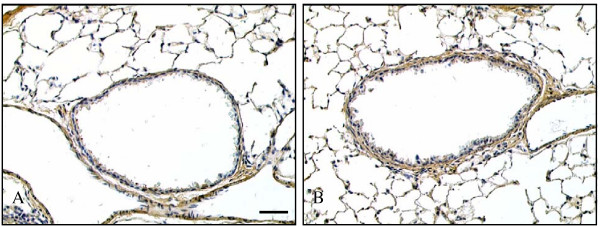
**The effect of ovalbumin sensitisation and challenge on airway sub-epithelial deposition of type III collagen. **Immunostaining of representative saline sensitised/challenged **(A)**and ovalbumin sensitised/challenged **(B) **airways for type III collagen 12 days after final challenge. In control lungs staining was localised to the alveolar septa, airway and vessel walls. Airway sub-epithelial staining was increased following ovalbumin sensitisation/ challenge. Scale bar represents 50 μm.

To confirm that the changes in MSB staining reflected changes in sub-epithelial collagen deposition we immunostained sections for type III collagen and quantified the area of sub-epithelial staining. Type III collagen showed a similar localisation pattern to that of the blue staining of MSB (figure 4). The area of sub-epithelial type III collagen staining in saline sensitised/saline challenged mice was 2.26 ± 0.14 μm^2^/μm (n = 8) and 2.92 ± 0.16 μm^2^/μm (n = 11) in ovalbumin sensitised/challenged mice (p < 0.01). This increase of approximately 30% was similar to the increase observed in sections from the same groups stained with MSB.

#### Increased sub-epithelial proteoglycan deposition

**Figure 5:**
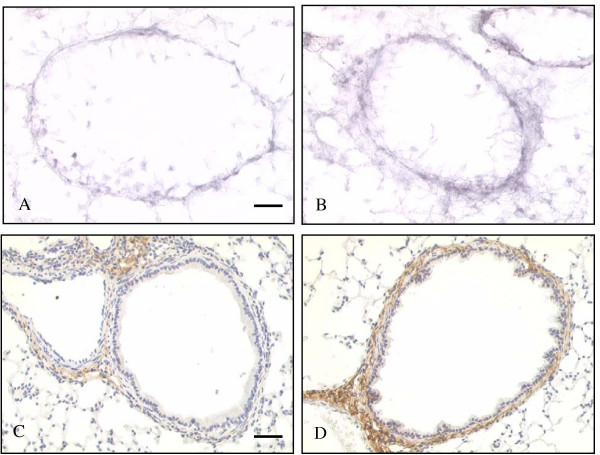
**The effect of ovalbumin sensitisation and challenge on airway sub-epithelial proteoglycan and decorin deposition. **Cupromeronic Blue staining of representative airways from **(A) **control and **(B) **ovalbumin sensitised/challenged airways 12 days after final challenge. The reticular pattern of staining is seen particularly around the airway circumference in a distribution corresponding to the sub-epithelial layer. Staining is greater in ovalbumin sensitised/challenged airways. Decorin immunostaining of representative airways from **(C) **control and **(D) **ovalbumin sensitised/challenged airways at the same time point. Immunostaining is concentrated in the walls of airways and vessels and is not seen in the lung parenchyma. Immunostaining is greater in ovalbumin sensitised/challenged airways and vessels. Scale bars represent 50 μm.

**Figure 6:**
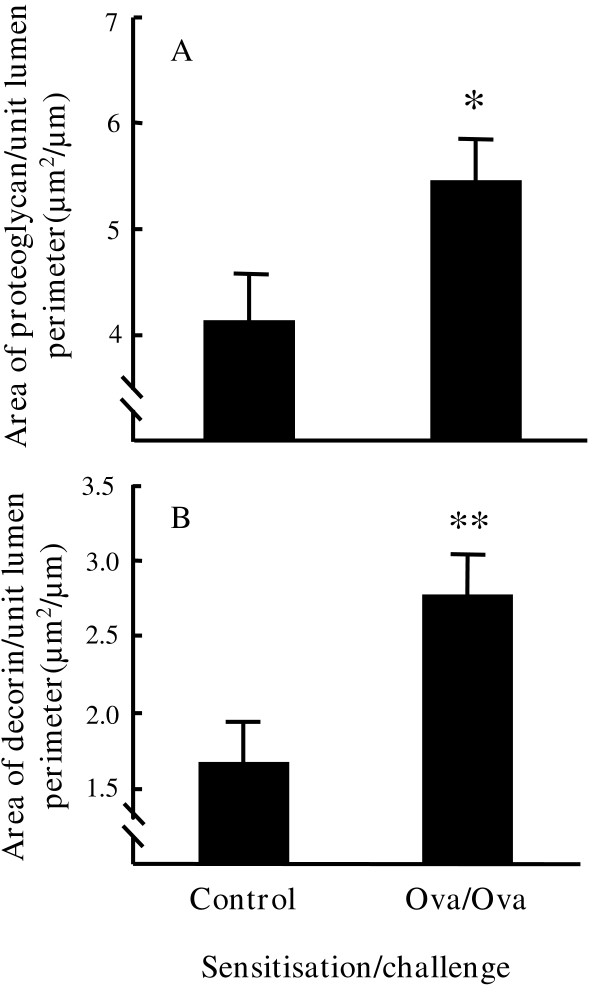
**The effect of ovalbumin sensitisation/challenge on sub-epithelial total proteoglycans and decorin. (A) **The area of sub-epithelial proteoglycan/unit lumen perimeter was quantitated using image analysis of Cupromeronic Blue stained sections. The numbers of mice/total airways analysed per group were: Control 9/32 and Ova/Ova 10/52. **(B) **The area of sub-epithelial decorin/unit lumen perimeter was quantitated using decorin immunostaining. The numbers of mice/total airways analysed per group were: Control 7/40 and Ova/Ova 10/70. * p < 0.05, ** p < 0.01.

Cupromeronic Blue staining demonstrated a reticular pattern of proteoglycan deposition beneath the epithelial layer (figure 5A). Staining was usually continuous around the airway circumference and was also seen in the lung parenchyma to a lesser degree. Increased staining was seen in ovalbumin sensitised/ovalbumin challenged mice compared with controls (figure 5A and 5B). Sub-epithelial proteoglycan/unit lumen perimeter was 4.13 ± 0.44 μm^2^/μm in control mice and 5.46 ± 0.39 μm^2^/μm in ovalbumin sensitised/challenged mice, corresponding to a 32% increase in proteoglycans (p < 0.05) (figure 6).

#### Increased sub-epithelial decorin deposition

In control airways staining for decorin was weak with stronger staining associated with blood vessels (figure 5C). In contrast, in the airways of ovalbumin sensitised/challenged mice decorin immunohistochemistry demonstrated a well-localised, intense band of staining beneath the epithelium (figure 5D). Immunostaining was not seen elsewhere in lung sections. Sub-epithelial decorin/unit lumen perimeter was 1.66 ± 0.27 μm^2^/μm in control mice and 2.76 ± 0.28 μm^2^/μm in ovalbumin sensitised/challenged mice. This equates with an increase of 66% in decorin in ovalbumin sensitised/challenged mice (p < 0.01) (figure 6).

## Discussion

Using a modified ovalbumin sensitisation/challenge protocol in a mouse strain often used for the generation of transgenics and knockouts, many of the features of airway remodelling have been reproduced. At 24 hours after final challenge, epithelial changes similar to those found in acute asthma are seen. There is epithelial cell loss, mucous cell metaplasia and inflammatory cell infiltration of the airway wall. This model, in contrast to several others published in the literature, has the advantage of not producing any obvious parenchymal changes. 12 days after final challenge, the acute inflammatory changes have largely resolved and increased deposition of sub-epithelial collagen and proteoglycans, including decorin, are demonstrated.

The current protocol is based on the widely used ovalbumin sensitisation and challenge method [25,26,37,38] using two intra-peritoneal sensitising injections and six daily intra-tracheal challenges of ovalbumin. The doses of ovalbumin for sensitisation and challenge fall within the ranges used in previous studies but the timings have been optimised to produce reproducible airway remodelling with increased and persistent deposition of extra-cellular matrix proteins including collagens and proteoglycans. Challenges were given by direct intra-tracheal instillation rather than by aerosol as this may more accurately control the dose of ovalbumin received by each mouse. Intra-tracheal instillation rather than aerosol inhalation may also have contributed to the reduction in parenchymal effects seen in this study.

We found similar increases in collagen deposition 12 and 28 days after final challenge when we analysed all airways together. However, when airways were sub-divided into groups with lumen perimeters of greater or less than 1000 μm the difference remained significant for larger airways at both time points but was only significant in smaller airways at the later time. A similar study in rats also found significant changes in smaller airways only at later times [39]. The reason for this is unclear but could be due to remodelling occurring more slowly in smaller airways, although further studies would be required to confirm this. The studies of Palmans and co-workers in rats categorised airway size according to basement membrane length and divided airways into groups with basement membrane length ≤1000 μm, >1000 μm and ≤2000 μm, and >2000 μm. In the present study with mice, airway size was based on the lumen perimeter as this could be measured more accurately with the stains used. Both basement membrane length and lumen perimeter measurements have previously been validated as markers of airway size that are independent of lung volume [40]. In our study very few airways had a perimeter of greater than 2000 μm. Therefore, airways were grouped as ≤1000 μm or >1000 μm.

The number of challenges varies dramatically between different published protocols used to induce airway remodelling. Two opposing theoretical considerations exist. In favour of a greater number of challenges is the, essentially intuitive, belief that remodelling only results from chronic, repetitive injury and repair [26,39]. In favour of fewer challenges is the risk of immunological tolerance developing as more challenges are given. This is not just a hypothetical risk as it has been demonstrated that prolonged exposure of mice to intra-nasal or aerosolised ovalbumin results in peripheral CD4(+) T-cell unresponsiveness, reduced ovalbumin-specific IgE production and suppression of airway inflammation [41,42]. We therefore used the minimum number of challenges that produced a significant and reproducible increase in sub-epithelial matrix.

Allergic airways disease is generally thought of as a Th2 driven response and most previous models of ovalbumin induced airway remodelling in mice have been developed in the BALB/c strain which has a strong Th2 response. The C57BL/6 and SV129 background strains do not generally exhibit strong Th2 responses [43] and may therefore not be ideal in some respects. However, the C57BL/6 strain has previously been used in studies of ovalbumin-induced airway remodelling [44] and the SV129 strain exhibits similar fibroproliferative responses to those of C57BL/6 in other models of fibrogenesis. Whilst we have not assessed the Th1/Th2 balance in the current studies, the fact that significantly increased and sustained deposition of sub-epithelial extracellular matrix proteins was observed, suggests that a strong Th2 response may not be critical to the development of airway remodelling. Further studies would be required to confirm this.

The sensitivity of different mouse strains is a considerable problem when modelling the various pathologic features of asthma including airway remodelling [29,30]. This has lead to the development of a multitude of ovalbumin sensitisation and challenge protocols in order to optimise the modelling of particular features of asthma in different mouse strains. The protocol developed in this study has used mixed strain mice, which are commonly used in the generation of genetically engineered mice and is commercially available. Ideally, genetically engineered mice should be backcrossed to a pure strain. However, due to constraints of time and cost, this is often not achieved. We therefore believe the model described here is readily reproducible and will be useful for studies utilising genetically engineered mice to investigate mechanisms involved in airway remodelling.

An important feature of this study is the use of digital image analysis for the quantification of sub-epithelial extra-cellular matrix deposition. Previous studies in mice have used a variety of histological methods including projection of airway images onto grids and point-counting [25], measurement of airway wall thickness with a microscope eyepiece reticle [32]and measurement of immunostaining intensity from black and white photomicrographs [31]. Other studies have attempted to quantitate airway extra-cellular matrix deposition using different techniques. These include measurement of hydroxyproline in whole lung [30,33] or in micro-dissected airways [45], and measurement of bronchoalveolar lavage fluid (BALF) cellular fibronectin content [37]. Significant disadvantages exist with each of these methods. Measurements derived from whole lung or BALF cannot be airway specific and this problem will be exacerbated in models that induce significant parenchymal changes. Accurate micro-dissection of airways as small as those found in the mouse is extremely difficult, particularly when we are concerned with a layer as thin and variable as the *lamina reticularis*. Computerised image analysis of stained sections has a number of advantages. It is reproducible between observers and allows accurate determination of extra-cellular matrix in the sub-epithelial layer of each airway. Anatomically, murine airways are frequently associated with other airways and blood vessels. Use of computerised image analysis allows individual airways to be isolated from surrounding structures. This facilitates selection of airway from non-airway matrix. The technique is also highly adaptable, allowing use of different stains to quantify different extra-cellular matrices.

The increase in sub-epithelial collagen in ovalbumin sensitised/challenged mice that we found using six consecutive challenges over six days is similar to that observed in other acute [25] and more chronic (3–12 weeks) [26,28,30,33] ovalbumin challenge studies. For example Blyth and co-workers observed a 55% increase in sub-epithelial reticulin staining after 6 ovalbumin challenges, three days apart, which was maintained for 50 days [25]. Similarly, Temelkovski and co-workers described a 55% increase in the thickness of the sub-epithelial layer after three ovalbumin challenges/week for 4 weeks, with further increases if challenges were continued for up to 8 weeks [26]. Furthermore, these changes are of a similar order of magnitude to those observed in the airways of asthmatics.

To further confirm that the sub-epithelial area of blue staining measured from MSB stained sections truly reflected an increase in collagen deposition we also immunostained sections for type III collagen. The percentage increases in the area of staining obtained for type III collagen and MSB staining following ovalbumin sensitisation/challenge were very similar. However, the values for type III collagen were about one third of those obtained from the MSB sections. This most likely reflects the difference between staining specifically for type III collagen and MSB, which is thought to stain all fibrillar collagens. Types I and III collagens represent greater than 95% of total lung collagen with type III collagen contributing approximately 30% of this total [46]. The proportional area of staining for type III collagen compared with the total blue MSB stained area is therefore consistent with the distribution of collagen types in the lung.

Previous studies examining increased sub-epithelial extra-cellular matrix deposition in murine models of airway remodelling have measured changes in collagen [28,32,45,48] and BAL cellular fibronectin [37,49]. Similarly, airway collagen and fibronectin have been measured in a rat model [39]. A unique feature of this study, therefore, is its application to measurement of increased sub-epithelial proteoglycans, including decorin.

The proteoglycans lumican, biglycan, versican, hyaluronan and decorin have been identified in biopsy studies of normal and asthmatic human airways. Lumican, biglycan, and versican immunostaining is predominantly found in the sub-epithelial layer of control airways [10]. Versican and hyaluronan are also localised around and internal to airway smooth muscle bundles [50]. Data on decorin immunostaining in controls are conflicting. Minimal airway decorin was demonstrated in one study [10], whereas extensive sub-epithelial staining was apparent in another [51]. In this study we show a similar sub-epithelial localisation of proteoglycan staining in mouse airways. We also show that decorin is localised to airway sub-epithelial extra-cellular matrix in mice as described previously for human [51], bovine [52] and rat [53] airways, and is increased following ovalbumin sensitisation and challenge. Since this part of the study used only saline sensitised/saline challenged animals as a control we cannot exclude the possibility that increased proteoglycan deposition is due to non-specific effects of instilling large amounts of ovalbumin into the airways. However, this seems unlikely given the demonstrated lack of effect of ovalbumin on collagen deposition in the saline sensitised/ovalbumin challenged mice (figures 2 and 3) and the evidence for increased sub-epithelial proteoglycan deposition in asthmatic airways.

Lumican, biglycan and versican are increased in the sub-epithelial layer of asthmatic airways compared with controls [10,24]. In our study, Cupromeronic Blue staining of proteoglycans was increased in ovalbumin sensitised/challenged mice with a similar sub-epithelial localisation to that observed in asthmatic airways, suggesting this is a good model of the changes observed in humans [51].

The finding of increased proteoglycans in the sub-epithelial layer of the airways of asthmatics and ovalbumin sensitised/challenged mice is of particular interest. Proteoglycans have an important role in determining organisation of collagen [54]. Decorin, particularly, is an important regulator of collagen fibril assembly [55,56]. Proteoglycans bind growth factors relevant to extra-cellular matrix deposition in the sub-epithelial layer. Perlecan binds specifically to fibroblast growth factor-7 [57]. A number of proteoglycans bind transforming growth factor-β, including decorin, biglycan and fibromodulin [58]. The increased immunostaining for decorin observed in ovalbumin sensitised/challenged mice is consistent with its sub-epithelial co-localisation with increased extra-cellular matrix, and TGF-β which has previously been shown to be increased [28]. The increase in sub-epithelial proteoglycans may be an important factor in the development of sub-epithelial airway remodelling.

## Conclusion

This animal model reproduces many of the features of airway remodelling found in asthma and allows accurate and reproducible measurement of sub-epithelial extra-cellular matrix deposition. As far as we are aware this is the first demonstration of increased sub-epithelial proteoglycans in an animal model of airway remodelling. Proteoglycans and collagen are increased by a similar magnitude to that demonstrated in asthmatics, providing a useful model of this aspect of the pathology. The properties of these matrix components suggest that they will have direct and indirect effects on airway function and the regulation of airway remodelling. The model will be invaluable in the assessment of the molecular mechanisms contributing to sub-epithelial airway remodelling and of agents to modulate such remodelling.

## Authors' contributions

AKR played a major role in the design of the study, acquisition, analysis and interpretation of data and drafting the manuscript. SEB contributed to the acquisition of data and development of the image analysis methods. GJL contributed to the interpretation of data. RJM conceived and coordinated the study, participated in the design of the study, analysis and interpretation of data and drafting the manuscript. All authors read and approved the final manuscript
